# 4-Meth­oxy-4-methyl-6-phenyl-1,3-diazinane-2-thione

**DOI:** 10.1107/S1600536812044662

**Published:** 2012-11-03

**Authors:** Seranthimata Samshuddin, Badiadka Narayana, Hemmige S. Yathirajan, Thomas Gerber, Eric Hosten, Richard Betz

**Affiliations:** a Mangalore University, Department of Studies in Chemistry, Mangalagangotri 574 199, India; b University of Mysore, Department of Studies in Chemistry, Manasagangotri, Mysore 570 006, India; c Nelson Mandela Metropolitan University, Summerstrand Campus, Department of Chemistry, University Way, Summerstrand, PO Box 77000, Port Elizabeth, 6031, South Africa

## Abstract

In the title pyrimidine derivative, C_12_H_16_N_2_OS, the tetra­hydro­pyrimidine ring adopts an envelope conformation with the C atom of the methyl­ene –CH_2_– group as the flap. In the crystal, N—H⋯O and N—H⋯S hydrogen bonds connect mol­ecules into undulating sheets perpendicular to the *a* axis.

## Related literature

For the pharmacological importance of pyrimidines, see: Selvam *et al.* (2012 ▶); Gupta *et al.* (2010 ▶); Lagoja (2005 ▶). For the crystal structures of related compounds, see: Kant *et al.* (2012 ▶); Fun *et al.* (2012 ▶); Betz *et al.* (2012 ▶). For puckering analysis of six-membered rings, see: Cremer & Pople (1975 ▶); Boeyens (1978 ▶). For graph-set analysis of hydrogen bonds, see: Etter *et al.* (1990 ▶); Bernstein *et al.* (1995 ▶).
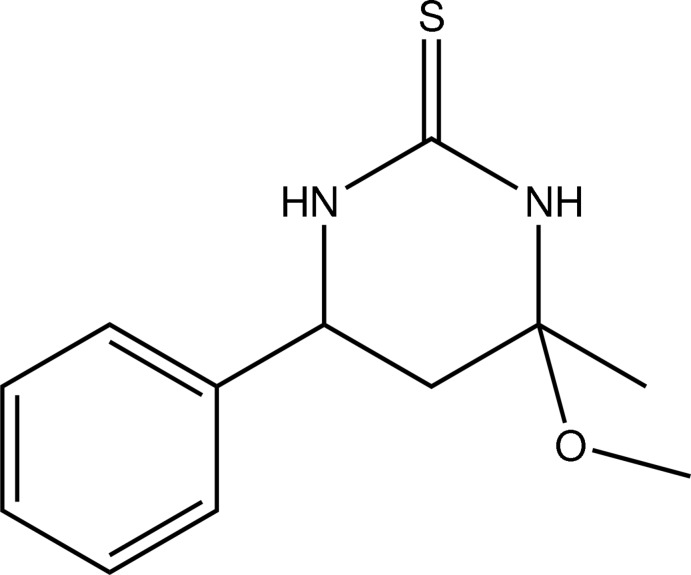


## Experimental

### 

#### Crystal data

C_12_H_16_N_2_OS
*M* *_r_* = 236.33
Monoclinic, 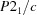

*a* = 10.1894 (3) Å
*b* = 14.6889 (4) Å
*c* = 9.2026 (2) Å
β = 111.719 (1)°
*V* = 1279.58 (6) Å^3^
*Z* = 4
Mo *K*α radiation
μ = 0.24 mm^−1^
*T* = 200 K
0.47 × 0.41 × 0.33 mm

#### Data collection

Bruker APEXII CCD diffractometer
Absorption correction: multi-scan (*SADABS*; Bruker, 2008 ▶) *T*_min_ = 0.898, *T*_max_ = 0.926
12126 measured reflections
3173 independent reflections
2876 reflections with *I* > 2σ(*I*)
*R*_int_ = 0.011

#### Refinement

*R*[*F* ^2^ > 2σ(*F* ^2^)] = 0.032
*wR*(*F* ^2^) = 0.089
*S* = 1.07
3173 reflections
155 parameters
H atoms treated by a mixture of independent and constrained refinement
Δρ_max_ = 0.33 e Å^−3^
Δρ_min_ = −0.23 e Å^−3^

### 

Data collection: *APEX2* (Bruker, 2010 ▶); cell refinement: *SAINT* (Bruker, 2010 ▶); data reduction: *SAINT*; program(s) used to solve structure: *SHELXS97* (Sheldrick, 2008 ▶); program(s) used to refine structure: *SHELXL97* (Sheldrick, 2008 ▶); molecular graphics: *ORTEP-3* (Farrugia, 1997 ▶) and *Mercury* (Macrae *et al.*, 2008 ▶); software used to prepare material for publication: *SHELXL97* and *PLATON* (Spek, 2009 ▶).

## Supplementary Material

Crystal structure: contains datablock(s) I, global. DOI: 10.1107/S1600536812044662/lh5545sup1.cif

Supplementary material file. DOI: 10.1107/S1600536812044662/lh5545Isup2.cdx

Structure factors: contains datablock(s) I. DOI: 10.1107/S1600536812044662/lh5545Isup3.hkl

Supplementary material file. DOI: 10.1107/S1600536812044662/lh5545Isup4.cml

Additional supplementary materials:  crystallographic information; 3D view; checkCIF report

## Figures and Tables

**Table 1:** Hydrogen-bond geometry (Å, °)

*D*—H⋯*A*	*D*—H	H⋯*A*	*D*⋯*A*	*D*—H⋯*A*
N1—H1⋯S1^i^	0.862 (15)	2.503 (16)	3.3527 (9)	168.8 (12)
N2—H2⋯O1^ii^	0.841 (15)	2.076 (16)	2.8863 (12)	161.7 (13)

Symmetry codes: (i) 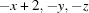; (ii) 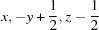.

**Fig. 1.**
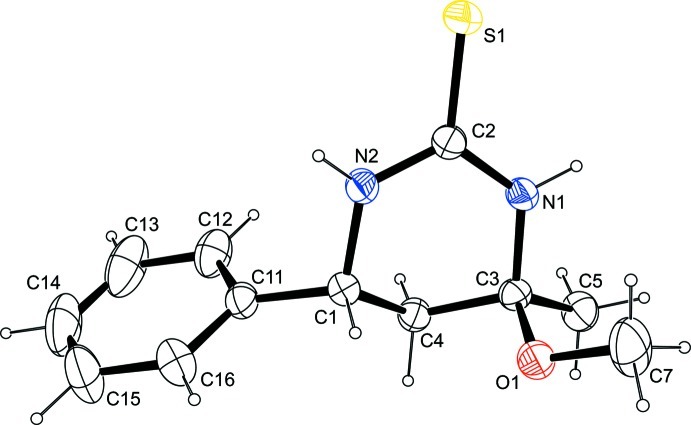
The molecular structure of the title compound, with anisotropic displacement ellipsoids drawn at the 50% probability level.

**Fig. 2.**
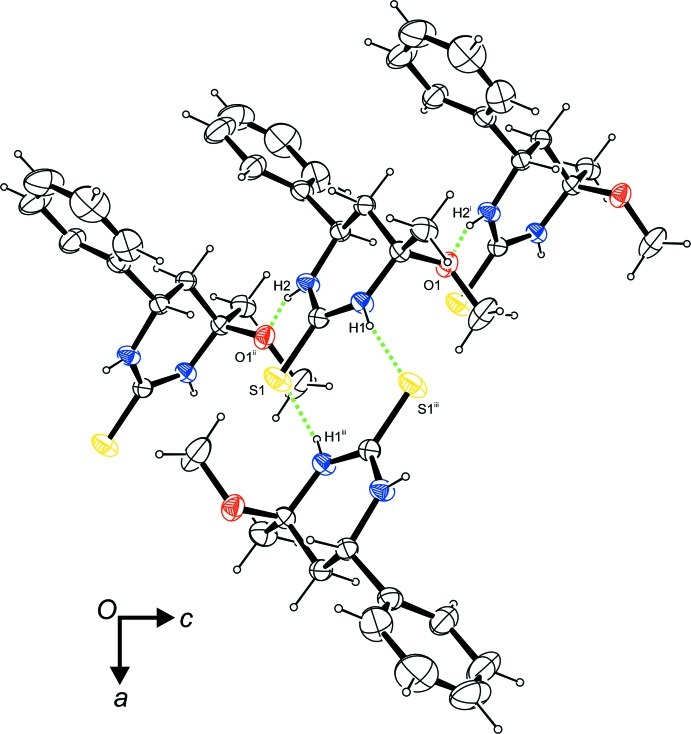
The hydrogen bond motif (dashed lines), viewed along [0 1 0]. Symmetry operators: (i) x, -y + 1/2, z + 1/2; (ii) x, -y + 1/2, z - 1/2; (iii) -x + 2, -y, -z.

## supplementary crystallographic information

### Comment

Pyrimidine and its derivatives exhibit remarkable pharmacological activities including anticonvulsant, antiinflammatory, antibacterial, antifungal, antiviral and anticancer properties (Selvam *et al.*, 2012; Gupta *et al.*, 2010). They constitute important building blocks of natural biologically active compounds like nucleic acids, several vitamins, coenzymes, purines and some marine microorganisms (Lagoja, 2005). Pyrimidine and its derivatives form a component in a number of marketed drugs including flucytosine (antimycotic), floxuridine (antimetabolite), ambrisentan (endothelin receptor antagonist), fluorouracil (antimetabolite), pyrimethamine (antimalarial), piribedil (antiparkinsonian), minoxidil (antihypertensive), carmofur (antineoplastic), bosentan (endothelin receptor antagonist) and many more. The crystal structures of some pyrimidine derivatives such as 2-[3,5-bis(4-methoxyphenyl)-4,5- dihydro-1*H*-pyrazol-1-yl]-4,6-bis(4-methoxyphenyl)pyrimidine (Kant *et al.*, 2012) and 2-[3,5-bis(4-fluorophenyl)-4,5-dihydro-1*H*-pyrazol-1-yl]- 4,6-bis(4-fluorophenyl)pyrimidine (Fun *et al.*, 2012) have been reported. In view of the pharmacological importance of pyrimidine derivatives and in continuation of our work on synthesis of pyrimidine derivatives (Betz *et al.*, 2012), we have determined the crystal structure of the title compound, 4-methoxy-4-methyl-6-phenyl-1,3-diazinane-2-thione.

According to a puckering analysis (Cremer & Pople, 1975; Boeyens, 1978), the tetrahydropyrimidine ring adopts an ^5^*E* conformation with atom C4 as the flap (^C(4)^*E*). The aromatic substituent is found in a nearly perpendicular conformation with respect to the tetrahydropyrimidine ring with the least-squares planes defined by the intracyclic atoms of the phenyl group on the one hand and the five essentially planar atoms [C1/C2/C3/N1/N2] of the 1,3-diazacyclohexane ring on the other hand intersecting at an angle of 74.95 (7) ° (Fig. 1).

In the crystal, N–H···O and N–H···S hydrogen bonds connect molecules into undulating sheets perpendicular to the crystallographic *a* axis. In terms of graph-set analysis (Etter *et al.*, 1990; Bernstein *et al.*, 1995), the descriptor for these contacts is *C*^1^_1_(6)*R*^2^_2_(8) on the unary level. Metrical parameters as well as information about the symmetry of these contacts are summarized in Table 1. The shortest intercentroid distance between two aromatic systems is 4.9991 (9) Å (Fig. 2).

### Experimental

To a mixture of benzylidene acetone (1.46 g, 0.01 mol) and thiourea (1.1 g, 0.015 mol) in methanol (20 ml), sodium methoxide solution (1 ml) was added and the batch was refluxed for 8 h. The precipitate formed was collected by filtration and dried, yield: 82%. The single crystal was grown from a DMF solution of the title compound by slow evaporation at room temperature.

### Refinement

Carbon-bound H atoms were placed in calculated positions (C—H 0.95 Å for aromatic carbon atoms, C—H 0.98Å for methyl groups, C—H 0.99 Å for the methylene group and C—H 1.00 Å for the methine group) and were included in the refinement in the riding model approximation, with *U*(H) set to 1.2*U*_eq_(C). The H atoms of the methyl groups were allowed to rotate with a fixed angle around the C—C bond to best fit the experimental electron density (HFIX 137 in the *SHELX* program suite (Sheldrick, 2008), with *U*(H) set to 1.5*U*_eq_(C). Both nitrogen-bound H atoms were located on a difference Fourier map and refined freely.

### Crystal data

**Table:**

C_12_H_16_N_2_OS	*F*(000) = 504
*M**_r_* = 236.33	*D*_x_ = 1.227 Mg m^−^^3^
Monoclinic, *P*2_1_/*c*	Melting point: 538 K
Hall symbol: -P 2ybc	Mo *K*α radiation, λ = 0.71073 Å
*a* = 10.1894 (3) Å	Cell parameters from 8597 reflections
*b* = 14.6889 (4) Å	θ = 2.8–28.3°
*c* = 9.2026 (2) Å	µ = 0.24 mm^−^^1^
β = 111.719 (1)°	*T* = 200 K
*V* = 1279.58 (6) Å^3^	Block, yellow
*Z* = 4	0.47 × 0.41 × 0.33 mm

### Data collection

**Table:**

Bruker APEXII CCD diffractometer	3173 independent reflections
Radiation source: fine-focus sealed tube	2876 reflections with *I* > 2σ(*I*)
Graphite monochromator	*R*_int_ = 0.011
φ and ω scans	θ_max_ = 28.3°, θ_min_ = 2.2°
Absorption correction: multi-scan (*SADABS*; Bruker, 2008)	*h* = −13→13
*T*_min_ = 0.898, *T*_max_ = 0.926	*k* = −19→18
12126 measured reflections	*l* = −11→12

### Refinement

**Table:**

Refinement on *F*^2^	Primary atom site location: structure-invariant direct methods
Least-squares matrix: full	Secondary atom site location: difference Fourier map
*R*[*F*^2^ > 2σ(*F*^2^)] = 0.032	Hydrogen site location: inferred from neighbouring sites
*wR*(*F*^2^) = 0.089	H atoms treated by a mixture of independent and constrained refinement
*S* = 1.07	*w* = 1/[σ^2^(*F*_o_^2^) + (0.045*P*)^2^ + 0.354*P*] where *P* = (*F*_o_^2^ + 2*F*_c_^2^)/3
3173 reflections	(Δ/σ)_max_ < 0.001
155 parameters	Δρ_max_ = 0.33 e Å^−^^3^
0 restraints	Δρ_min_ = −0.23 e Å^−^^3^

### Fractional atomic coordinates and isotropic or equivalent isotropic displacement parameters (Å^2^)

**Table:**

	*x*	*y*	*z*	*U*_iso_*/*U*_eq_	
S1	0.93913 (3)	0.10930 (2)	−0.17540 (4)	0.03661 (10)	
O1	0.83361 (8)	0.14192 (5)	0.27715 (9)	0.03043 (18)	
N1	0.84364 (9)	0.06949 (6)	0.04949 (10)	0.02460 (18)	
H1	0.9003 (15)	0.0236 (10)	0.0685 (16)	0.035 (3)*	
N2	0.75092 (9)	0.19789 (6)	−0.09623 (11)	0.02608 (18)	
H2	0.7641 (14)	0.2391 (10)	−0.1528 (17)	0.034 (3)*	
C1	0.66034 (10)	0.21995 (7)	−0.00883 (11)	0.02360 (19)	
H1A	0.7138	0.2607	0.0806	0.028*	
C2	0.83890 (10)	0.12684 (7)	−0.06586 (11)	0.0237 (2)	
C3	0.76328 (10)	0.08187 (7)	0.14971 (11)	0.02301 (19)	
C4	0.62693 (10)	0.13127 (7)	0.05627 (12)	0.0250 (2)	
H4A	0.5735	0.1445	0.1244	0.030*	
H4B	0.5676	0.0921	−0.0308	0.030*	
C5	0.73786 (12)	−0.01063 (8)	0.20866 (14)	0.0331 (2)	
H5A	0.6847	−0.0493	0.1195	0.050*	
H5B	0.6837	−0.0029	0.2764	0.050*	
H5C	0.8288	−0.0394	0.2681	0.050*	
C7	0.97628 (15)	0.12213 (10)	0.37158 (17)	0.0499 (4)	
H7A	1.0353	0.1282	0.3088	0.075*	
H7B	0.9829	0.0597	0.4114	0.075*	
H7C	1.0090	0.1648	0.4596	0.075*	
C11	0.52944 (11)	0.26970 (8)	−0.11439 (12)	0.0284 (2)	
C12	0.43381 (12)	0.22891 (10)	−0.24745 (15)	0.0396 (3)	
H12	0.4503	0.1688	−0.2747	0.048*	
C13	0.31340 (14)	0.27604 (13)	−0.34123 (19)	0.0581 (4)	
H13	0.2484	0.2479	−0.4325	0.070*	
C14	0.28830 (16)	0.36287 (14)	−0.3024 (2)	0.0655 (5)	
H14	0.2053	0.3943	−0.3656	0.079*	
C15	0.3827 (2)	0.40383 (13)	−0.1729 (2)	0.0688 (5)	
H15	0.3657	0.4641	−0.1467	0.083*	
C16	0.50400 (17)	0.35781 (10)	−0.07860 (18)	0.0479 (3)	
H16	0.5696	0.3871	0.0108	0.057*	

### Atomic displacement parameters (Å^2^)

**Table:**

	*U*^11^	*U*^22^	*U*^33^	*U*^12^	*U*^13^	*U*^23^
S1	0.04262 (18)	0.03902 (18)	0.03912 (17)	0.01582 (12)	0.02784 (14)	0.01265 (11)
O1	0.0299 (4)	0.0309 (4)	0.0259 (4)	0.0042 (3)	0.0050 (3)	−0.0048 (3)
N1	0.0276 (4)	0.0221 (4)	0.0272 (4)	0.0053 (3)	0.0137 (3)	0.0038 (3)
N2	0.0264 (4)	0.0259 (4)	0.0299 (4)	0.0049 (3)	0.0150 (3)	0.0078 (3)
C1	0.0232 (4)	0.0244 (4)	0.0234 (4)	0.0032 (4)	0.0089 (4)	0.0013 (3)
C2	0.0229 (4)	0.0246 (5)	0.0238 (5)	0.0003 (3)	0.0090 (4)	0.0004 (3)
C3	0.0244 (4)	0.0228 (4)	0.0230 (4)	0.0003 (3)	0.0102 (4)	0.0008 (3)
C4	0.0220 (4)	0.0279 (5)	0.0260 (5)	0.0012 (4)	0.0099 (4)	0.0028 (4)
C5	0.0355 (5)	0.0280 (5)	0.0402 (6)	0.0011 (4)	0.0192 (5)	0.0092 (4)
C7	0.0379 (7)	0.0491 (8)	0.0437 (7)	0.0081 (6)	−0.0070 (6)	−0.0078 (6)
C11	0.0260 (5)	0.0318 (5)	0.0301 (5)	0.0062 (4)	0.0134 (4)	0.0082 (4)
C12	0.0298 (5)	0.0447 (7)	0.0385 (6)	−0.0028 (5)	0.0059 (5)	0.0109 (5)
C13	0.0289 (6)	0.0783 (11)	0.0543 (8)	−0.0064 (7)	0.0005 (6)	0.0294 (8)
C14	0.0392 (7)	0.0798 (12)	0.0785 (12)	0.0273 (8)	0.0227 (8)	0.0470 (10)
C15	0.0746 (12)	0.0558 (10)	0.0820 (12)	0.0406 (9)	0.0359 (10)	0.0253 (9)
C16	0.0579 (8)	0.0387 (7)	0.0479 (7)	0.0200 (6)	0.0205 (7)	0.0063 (6)

### Geometric parameters (Å, º)

**Table:**

S1—C2	1.6994 (10)	C5—H5B	0.9800
O1—C7	1.4206 (14)	C5—H5C	0.9800
O1—C3	1.4301 (12)	C7—H7A	0.9800
N1—C2	1.3420 (13)	C7—H7B	0.9800
N1—C3	1.4536 (12)	C7—H7C	0.9800
N1—H1	0.862 (15)	C11—C16	1.3835 (17)
N2—C2	1.3362 (13)	C11—C12	1.3873 (17)
N2—C1	1.4674 (12)	C12—C13	1.3949 (18)
N2—H2	0.841 (15)	C12—H12	0.9500
C1—C11	1.5159 (13)	C13—C14	1.374 (3)
C1—C4	1.5241 (14)	C13—H13	0.9500
C1—H1A	1.0000	C14—C15	1.364 (3)
C3—C5	1.5203 (14)	C14—H14	0.9500
C3—C4	1.5206 (13)	C15—C16	1.394 (2)
C4—H4A	0.9900	C15—H15	0.9500
C4—H4B	0.9900	C16—H16	0.9500
C5—H5A	0.9800		
			
C7—O1—C3	117.66 (9)	C3—C5—H5B	109.5
C2—N1—C3	124.02 (8)	H5A—C5—H5B	109.5
C2—N1—H1	118.5 (9)	C3—C5—H5C	109.5
C3—N1—H1	117.4 (9)	H5A—C5—H5C	109.5
C2—N2—C1	124.55 (8)	H5B—C5—H5C	109.5
C2—N2—H2	116.4 (10)	O1—C7—H7A	109.5
C1—N2—H2	117.2 (10)	O1—C7—H7B	109.5
N2—C1—C11	109.75 (8)	H7A—C7—H7B	109.5
N2—C1—C4	107.70 (8)	O1—C7—H7C	109.5
C11—C1—C4	113.10 (8)	H7A—C7—H7C	109.5
N2—C1—H1A	108.7	H7B—C7—H7C	109.5
C11—C1—H1A	108.7	C16—C11—C12	118.81 (11)
C4—C1—H1A	108.7	C16—C11—C1	119.76 (11)
N2—C2—N1	118.74 (9)	C12—C11—C1	121.43 (10)
N2—C2—S1	119.89 (8)	C11—C12—C13	120.09 (14)
N1—C2—S1	121.36 (8)	C11—C12—H12	120.0
O1—C3—N1	111.63 (8)	C13—C12—H12	120.0
O1—C3—C5	111.01 (8)	C14—C13—C12	120.37 (16)
N1—C3—C5	108.97 (8)	C14—C13—H13	119.8
O1—C3—C4	104.19 (8)	C12—C13—H13	119.8
N1—C3—C4	108.19 (8)	C15—C14—C13	119.86 (13)
C5—C3—C4	112.80 (8)	C15—C14—H14	120.1
C3—C4—C1	109.87 (8)	C13—C14—H14	120.1
C3—C4—H4A	109.7	C14—C15—C16	120.42 (16)
C1—C4—H4A	109.7	C14—C15—H15	119.8
C3—C4—H4B	109.7	C16—C15—H15	119.8
C1—C4—H4B	109.7	C11—C16—C15	120.43 (15)
H4A—C4—H4B	108.2	C11—C16—H16	119.8
C3—C5—H5A	109.5	C15—C16—H16	119.8
			
C2—N2—C1—C11	−151.62 (10)	N2—C1—C4—C3	53.96 (10)
C2—N2—C1—C4	−28.10 (13)	C11—C1—C4—C3	175.41 (8)
C1—N2—C2—N1	1.84 (15)	N2—C1—C11—C16	−117.76 (12)
C1—N2—C2—S1	−179.57 (8)	C4—C1—C11—C16	121.95 (12)
C3—N1—C2—N2	−2.99 (15)	N2—C1—C11—C12	62.03 (13)
C3—N1—C2—S1	178.44 (7)	C4—C1—C11—C12	−58.26 (13)
C7—O1—C3—N1	−52.81 (13)	C16—C11—C12—C13	−0.99 (18)
C7—O1—C3—C5	68.98 (13)	C1—C11—C12—C13	179.21 (11)
C7—O1—C3—C4	−169.33 (11)	C11—C12—C13—C14	−0.3 (2)
C2—N1—C3—O1	−83.72 (11)	C12—C13—C14—C15	1.1 (2)
C2—N1—C3—C5	153.33 (10)	C13—C14—C15—C16	−0.7 (3)
C2—N1—C3—C4	30.35 (13)	C12—C11—C16—C15	1.4 (2)
O1—C3—C4—C1	63.61 (10)	C1—C11—C16—C15	−178.77 (14)
N1—C3—C4—C1	−55.28 (10)	C14—C15—C16—C11	−0.6 (3)
C5—C3—C4—C1	−175.90 (8)		

### Hydrogen-bond geometry (Å, º)

**Table:**

*D*—H···*A*	*D*—H	H···*A*	*D*···*A*	*D*—H···*A*
N1—H1···S1^i^	0.862 (15)	2.503 (16)	3.3527 (9)	168.8 (12)
N2—H2···O1^ii^	0.841 (15)	2.076 (16)	2.8863 (12)	161.7 (13)

Symmetry codes: (i) −*x*+2, −*y*, −*z*; (ii) *x*, −*y*+1/2, *z*−1/2.
